# Policies and Practices of Cross-border Teleconsultation in Japan

**DOI:** 10.14789/ejmj.JMJ25-0016-P

**Published:** 2025-11-18

**Authors:** YAN YAN, TOSHIO NAITO

**Affiliations:** 1 Department of General Medicine, Juntendo University Faculty of Medicine, Tokyo, Japan; 1 Department of General Medicine, Juntendo University Faculty of Medicine, Tokyo, Japan; 2 Department of International Healthcare, Juntendo University Hospital, Tokyo, Japan; 2 Department of International Healthcare, Juntendo University Hospital, Tokyo, Japan

**Keywords:** teleconsultation, cross-border, inbound patient, medical tourism, COVID-19

## Abstract

Teleconsultation in Japan was initially introduced as a doctor-to-doctor or dentist-to-dentist (D-to-D) service, restricted to non-emergency, follow-up consultations for patients already under medical care and in stable condition. Following the onset of COVID-19 in February 2020 and the resulting restrictions on international travel, demand increased not only for first-time doctor-to-patient (D-to-P) consultations within Japan but also for cross-border teleconsultations. This study aimed to examine the regulatory evolution and implementation challenges of cross-border teleconsultation in Japan. While cross-border teleconsultation offers accessible and convenient care for international patients, broader implementation remains limited due to challenges such as inadequate international patient support systems and the absence of consistent international legal frameworks. The findings highlight the need to develop standardized international telemedicine frameworks and to establish robust communication channels among governments.

## Policy evolution of teleconsultation in Japan

Teleconsultation in Japan, initially referred to as “consultation using information and communication devices,” was first implemented as a doctor-to-doctor or dentist-to-dentist (D-to-D) service. This system enabled healthcare professionals to seek diagnostic or treatment advice from medical specialists. Over time, advancements in information technology and the growing need to provide healthcare in medically underserved areas led to the gradual adoption of doctor-to-patient or dentist-to-patient (D-to-P) teleconsultation.

On December 24, 1997, the Ministry of Health, Labour and Welfare (MHLW) issued a directive (hereafter referred to as the *1997 directive*) that laid the foundational principles for teleconsultation involving patients^1)^. For the first time, it was clarified that such practices did not violate Article 20 of the Medical Practitioners Act, which prohibits medical treatment and prescriptions without patient consultation^2)^. The *1997 directive* specified that physicians could lawfully obtain patients' health information remotely through information and communication technologies, thereby legitimizing teleconsultation. However, its use was limited to non-emergency, follow-up consultations for patients already under medical care and in stable condition; first-time consultations and emergency care were explicitly excluded.

Following the onset of COVID-19 in Japan in February 2020, healthcare resources were partially reallocated to manage the pandemic, while repeated “stay-at-home” advisories increased demand for remote medical services. In response, the MHLW issued a notice in April 2020 ─ *Time-limited and Special Policies Regarding the Use of Telephone and Other Information and Communication Devices During the COVID-19 Pandemic*^3)^. Significantly, this notice allowed first-time consultations to be conducted via telecommunication for the first time. Recognizing the effectiveness of teleconsultation during the pandemic, the Japanese government formally incorporated it into its broader healthcare digital transformation strategy in May 2023^4)^.

It is important to note that these policy changes primarily addressed domestic teleconsultation within Japan. While the pandemic accelerated the integration of telemedicine into the national healthcare system, it also disrupted access for international patients who had previously traveled to Japan for advanced medical care. As a result, how to accommodate the teleconsultation needs of overseas patients remains an unresolved issue and a critical area for future policy development.

## Needs for cross-border teleconsultation in Japan

The demand for cross-border teleconsultation in Japan predates the COVID-19 pandemic. Within the context of medical tourism, while some international visitors travel to Japan for comprehensive health examinations ─ such as the *Ningen Dock* ─ a substantial number seek advanced medical treatments. Prior to traveling, these patients often wish to understand who their attending physician will be and what the proposed treatment plan entails.

In Japan, most tertiary care institutions, including university hospitals, charge international patients without Japanese national health insurance a premium ranging from 100% to 400% of the base cost (i.e., the pre-insurance price) for the same medical services provided to insured Japanese citizens^5)^. As a result, inbound patients ─ particularly those seeking high-cost procedures such as surgery or long-term treatments like chemotherapy ─ must prepare for significant financial commitments. Moreover, patients with serious illnesses, such as cancer, often require complex travel arrangements and the support of accompanying family members. Understandably, these individuals seek detailed information in advance regarding their physician, treatment options, and the hospital environment. Online teleconsultation serves as an effective means of meeting these informational and preparatory needs.

Additionally, post-treatment follow-up presents a longstanding challenge for both international patients and Japanese healthcare providers. After completing treatment in Japan and returning home, many patients find it difficult to maintain regular follow-up visits due to time constraints and financial burdens. Frequent travel back to Japan is often impractical. This has contributed to a degree of reluctance among Japanese physicians to accept overseas patients. Teleconsultation, however, offers a practical solution ─ enabling continued communication and remote follow-up care between Japanese doctors and their international patients. In doing so, it supports continuity of care across borders and enhances the overall sustainability of cross-border medical services.

## Regulations on cross-border teleconsultation in Japan

Although online cross-border teleconsultation was practiced during the COVID-19 pandemic, it was not formally recognized in government policy until recently. In January 2024, MHLW revised its *Q&A on the Principles for the Appropriate Implementation of Online Medical Services* and, for the first time, explicitly addressed the issue of cross-border teleconsultation. The newly added Q&A item states (English translation)^6)^:

Q28: Are the Principles for the Appropriate Implementation of Online Medical Services applicable when a patient residing overseas receives medical services (including consultations, with or without online diagnosis or treatment) from a physician located in Japan?

A28: When medical services—such as consultations, diagnoses, or prescriptions ─ are provided by a physician located in Japan, Japanese laws and regulations, including the Medical Practitioners Act, the Medical Care Act, and the Principles for the Appropriate Implementation of Online Medical Services, apply ─ even if the patient is located overseas. However, providers must also ensure compliance with the relevant laws and regulations of the patient’s country of residence when delivering such online medical services.

This inclusion represents a significant milestone in the formal recognition of cross-border teleconsultation within Japan’s legal and medical framework. It not only clarifies the extraterritorial applicability of Japanese medical laws but also underscores the importance of complying with the legal and regulatory requirements of the patient’s country of residence.

## Practice of cross-border teleconsultation at a Japanese university hospital (Juntendo University Hospital)

Cross-border teleconsultation at Juntendo University Hospital emerged as a pragmatic response to the challenges posed by the COVID-19 pandemic. During this period, international travel was severely restricted by visa limitations and quarantine requirements, making it increasingly difficult for overseas patients to access medical care in Japan. These constraints significantly heightened the demand for remote medical consultations across borders.

**Figure 1:**
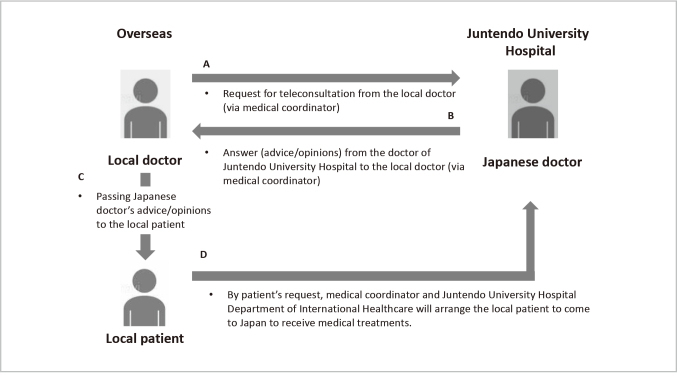
Diagram of teleconsultation in Juntendo University Hospital

In the absence of clear government policy on cross-border teleconsultation at the time, Juntendo University Hospital implemented a doctor-to-doctor (D-to-D) teleconsultation model (see Figure 1). This approach was intentionally designed to align with the principles set forth in the 1997 MHLW guidelines, which permitted first-time consultations exclusively between medical professionals. By adhering to this framework, the hospital maintained compliance with existing Japanese medical regulations while responding to the urgent needs of international patients during the pandemic.

## Benefits and challenges of cross-border teleconsultation in Japan

As with domestic teleconsultation, cross-border teleconsultation provides a convenient and accessible means for patients to receive medical advice and care remotely^7)^. For patients residing overseas, gaining a preliminary understanding of proposed treatments, potential outcomes, and possible side effects prior to traveling to Japan allows for more informed decision-making and better mental, financial, and logistical preparation. Importantly, it also enables patients to explore alternative treatment options and destinations, thereby enhancing patient autonomy.

International patients seeking advanced medical care in Japan often encounter language and cultural barriers, as well as substantial financial costs. The decision to pursue treatment abroad is rarely straightforward. In this context, cross-border teleconsultation serves as a valuable preliminary step ─ particularly for patients with complex or serious medical conditions.

Nevertheless, several challenges hinder the broader implementation of cross-border teleconsultation. Japanese physicians may receive incomplete or poorly translated medical information from international coordinators, which can impair accurate clinical assessment. As in other countries, concerns about legal liability and the risk of malpractice may cause Japanese doctors to hesitate in offering medical opinions or recommendations^8)^. Additionally, regulations governing the cross-border transmission of medical data vary significantly across jurisdictions^9)^. The lack of a clear international legal framework, coupled with limited institutional legal resources, poses a barrier for many Japanese medical institutions attempting to provide secure and compliant cross-border teleconsultation services.

## Author contributions

YY and TN conceptualized the study. YY conducted the majority of the literature review. NT provided overall supervision for the research project. YY drafted the initial version of the manuscript. All authors reviewed and approved the final manuscript. All authors meet the authorship criteria outlined by the International Committee of Medical Journal Editors (ICMJE).

## Conflicts of interest statement

The authors declare that there are no conflicts of interest.
